# PI(4,5)P_2_: signaling the plasma membrane

**DOI:** 10.1042/BCJ20220445

**Published:** 2022-11-11

**Authors:** Rachel C. Wills, Gerald R. V. Hammond

**Affiliations:** Department of Cell Biology, University of Pittsburgh School of Medicine, Pittsburgh, PA, U.S.A.

**Keywords:** lipid rafts, phospholipids, PIP2, PtdIns4, 5P2, signaling

## Abstract

In the almost 70 years since the first hints of its existence, the phosphoinositide, phosphatidyl-D-*myo*-inositol 4,5-bisphosphate has been found to be central in the biological regulation of plasma membrane (PM) function. Here, we provide an overview of the signaling, transport and structural roles the lipid plays at the cell surface in animal cells. These include being substrate for second messenger generation, direct modulation of receptors, control of membrane traffic, regulation of ion channels and transporters, and modulation of the cytoskeleton and cell polarity. We conclude by re-evaluating PI(4,5)P_2_’s designation as a signaling molecule, instead proposing a cofactor role, enabling PM-selective function for many proteins.

## Introduction

**Figure 1.**
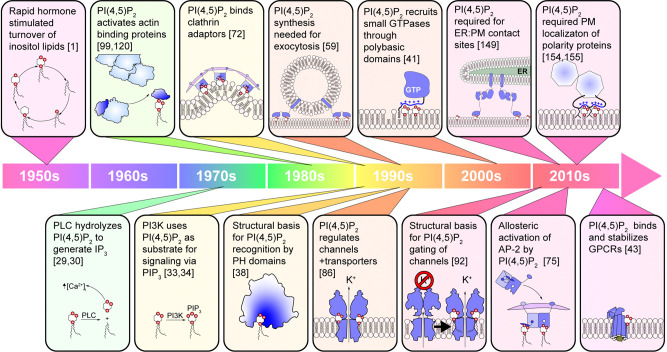
A timeline of several milestones in our understanding of PI(4,5)P_2_ function.

Phosphoinositides were first discovered in the 1950s as a class of phospholipid with a staggeringly rapid metabolic turnover [1]. Since then, the most abundant species, PI(4,5)P_2_, has been associated with a steadily growing list of cellular functions (see the timeline in Figure 1). As we will describe, these functions are legion and sundry; they have been the topic of many recent in-depth reviews [2–7]. However, a combined overview of the full gamut of PI(4,5)P_2­_ function has been lacking in contemporary literature. Our goal here is to provide the reader with a brief but broad summary of PI(4,5)P_2_ function in cell biology.

**Figure 2.**
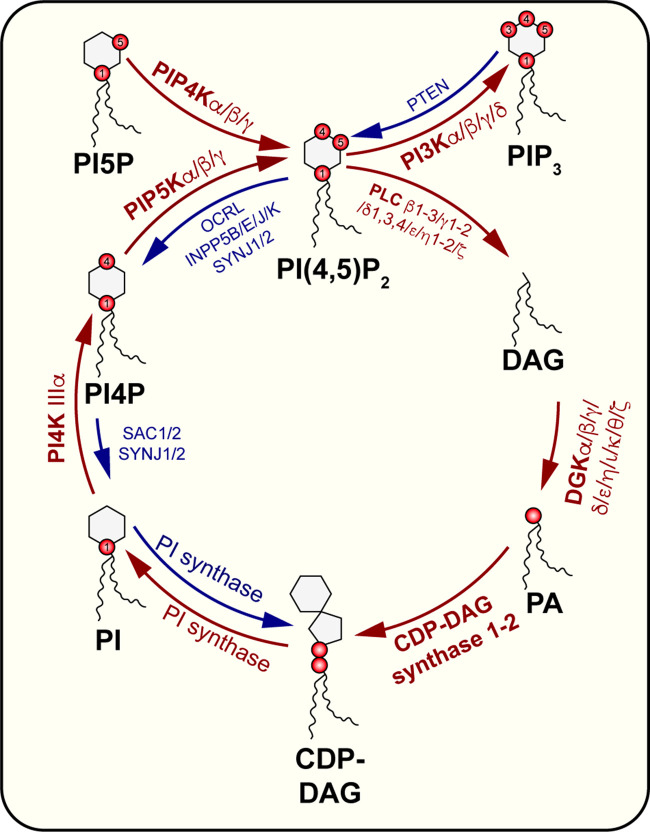
Synthesis and turnover of PI(4,5)P_2_. The major intermediate lipids species and the enzymes involved are indicated, restricted to those isoforms believed to be directly involved in PM PI(4,5)P_2_ synthesis.

The article will focus on the history and current understanding of PI(4,5)P_2_ regulation at the plasma membrane (PM). We do not discuss PI(4,5)P_2_ synthesis and turnover, though this is summarized in Figure 2. Excellent historical perspectives have covered the discovery of PI(4,5)P_2_ and its metabolism [8,9]. A notable feature of PI(4,5)P_2_ as a lipid in animal cells is that, like the other phosphoinositides, it seems to be very selectively endowed with *sn*-1 stearoyl (18:0) and *sn*-2 arachidonyl (20:4) tails. Intriguingly, despite making a rather large and disordered-domain preferring lipid, recent evidence suggests that the reason for this specific fatty acid profile is to enable ‘metabolic channeling’ of a reserved set of lipid intermediates for phosphoinositide synthesis and recycling [10,11].

A confluence of biochemical [12], cytochemical [13,14] and biosensor-based [15,16] approaches have revealed that most PI(4,5)P_2_, though not all, is found in the inner leaflet of the PM. It is important to note that we are still discovering critical cellular functions that are regulated by smaller, intracellular pools of PI(4,5)P_2_; recent examples have included the assembly of nuclear signaling complexes [17] and the regulation of peroxisomal fatty acid oxidation [18]. However, as we see in Figure 1, most of the lipid's functions have been associated with the PM. These PMs-selective functions are also those for which we have the most detailed mechanistic knowledge. Therefore, we will focus our discussion exclusively on this membrane.

Before detailing the many PM functions of PI(4,5)P_2_, it is worth enumerating the molecule in its native cellular environment. It is often remarked in the literature that PI(4,5)P_2_ is a minor or scarce lipid. We would argue that this depends on perspective. Certainly, PI(4,5)P_2_ makes up only 1–5% of total inositol lipid in cells [19–21], and the whole family is the least abundant of the phospholipids, accounting for ∼10% of glycerophospholipid [22]. So PI(4,5)P_2_ is expected to be merely 0.1–0.5% of total glycerophospholipid: scarce indeed. However, it is worth remembering that the confluence of empirical evidence over the last half century has indicated that, unlike most phospholipids, PI(4,5)P_2_ is heavily enriched in the inner leaflet of the PM. Therefore, the lipid has a much higher mole fraction here, perhaps accounting for up to 5% [23]. So, its local concentration in membranes can be high.

Another consideration is PI(4,5)P_2_’s abundance compared with the other molecules that it interacts with, often stoichiometrically: the proteins. Let's start by asking how many PI(4,5)P_2_ there are in the inner leaflet of the PM: assuming that the average leaflet of the bilayer can hold ∼1.5 × 10^6^ phospholipids/µm^2^ [21,24] and that 60% of that space is not occupied by membrane proteins [21] there is space for a total of ∼900 000 phospholipids/µm^2^ in the inner leaflet of the PM. Various PI(4,5)P_2_ densities have been experimentally determined and range from 4000 molecules/µm^2^ in a neuroblastoma cell line [25] to 34 000 molecules/µm^2^ in adult rat pinealocytes [21], or 0.4–4 mol%, resulting in an average of ∼20 000 PI(4,5)P_2_ molecules/µm^2^ [26], or 2%. Furthermore, estimating for the surface area of the PM at ∼2000 µm^2^ [27,28], it can be determined that there are anywhere between 8 × 10^6^ and 6.8 × 10^7^ PI(4,5)P_2_ molecules in the inner leaflet of the PM at any given time. Compare this to the median protein copy number of ∼3 × 10^4^ in typical cultured cells [29,30]. Even the most abundant structural proteins, such as components of the cytoskeleton, are present at ∼ 1 × 10^6^ copies per cell. PI(4,5)P_2_ outnumbers them all by an order of magnitude, even by the lower estimates of abundance. Put simply, PI(4,5)P_2_ may be a scarce lipid, but it is an exceptionally abundant regulatory molecule.

Given such a preponderance of PI(4,5)P_­2_ molecules in the PM, it is easy to see how it is able to bind the scores of effector proteins that it regulates. We will focus the majority of this article detailing the core classes of these interacting partners. Furthermore, we subdivide them by the major types of PM function they regulate: signaling, transport and structure.

## Signaling: substrate and cofactor

**Figure 3.**
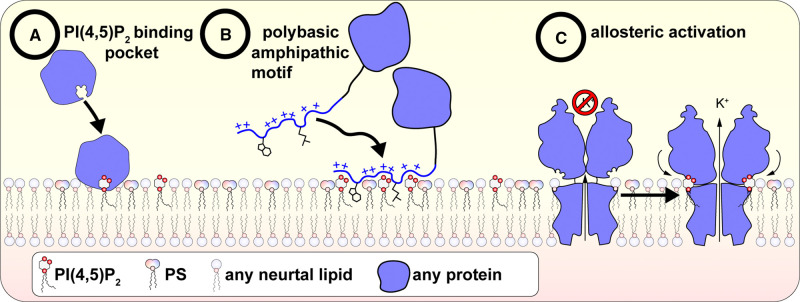
Mechanisms of protein regulation by PI(4,5)P_2_.

A key function of the PM in metazoans is to relay and amplify signals from impermeant, extracellular hormones and growth factors to the appropriate cellular response machinery. The seminal discovery of PI(4,5)P_2_ function was the realization that the lipid's hydrolysis by phospholipase C (PLC) in response to hormones stimulated cytosolic calcium entry [31], specifically from intracellular stores [32]. This rapid hydrolysis of PI(4,5)P_2_ by PLC also explained the unusually rapid turnover of PI(4,5)P_2_ and its metabolites that had initiated the study of the molecule over two decades earlier. At the time of writing, all of the human PLC isoforms (13 in total) have been identified with physiological functions being assigned [33], and an ever more sophisticated mechanistic knowledge of their regulation is being deduced [34]. The study of PLC activation also gave us the identification of the first highly selective, stoichiometric PI(4,5)P_2_ binding domain: the pleckstrin homology domain from PLCδ1 [35,36], unveiling a paradigm for PI(4,5)P_2_-dependent regulation (Figure 3A).

In some ways overtaking the discovery of PLC signaling was the realization that phosphoinositide 3-kinases (PI3Ks) utilize PI(4,5)P_2_ as a substrate to generate PIP_3_­ [37,38], a critically important second messenger in its own right. The PI3K pathway is now known to activate scores of downstream effector proteins and to play central functions in organismal growth control and immune activation [39]. Consequently, PI3Ks are becoming increasingly important targets for small molecule inhibitors in active clinical use, particularly in cancer [40]. In general, the importance of the PI3K and PLC pathways is now well established, ‘textbook’ knowledge. So, we will not dwell on them here.

Following this foundation as a substrate for second messenger generation, PI(4,5)P_2_ is now increasingly recognized as an enabling factor in many other signaling pathways at the PM, beyond those employing PLC or PI3K. Some of the first examples came from the large Ras superfamily of small GTPases, which are anchored to membranes by their C-termini. Specific PM targeting is often mediated by a C-terminal prenylation, with an adjacent polybasic stretch of residues that favor the PS-enriched, highly anionic inner leaflet of the PM [41,42]. PI(4,5)P_2_ is a critical component of this PM anionic lipid mix that allows specific PM targeting by these ubiquitous signaling proteins [43,44]. This non-selective, hydrophobic and electrostatic interaction with the membrane is another fundamental mechanism of lipid regulation (Figure 3B).

Beyond small GTPases, three class A heterotrimeric G-protein-coupled receptors (GPCRs) have been shown to bind to PI(4,5)P_2_ with high specificity, including adenosine A2A, β1 adrenergic and neurotensin receptor 1 [45]. Furthermore, the PI(4,5)P_2_ interacting residues of GPCRs were mapped to the cytosolic loops which link specific transmembrane (TM) helices together [45]. Moreover, this interaction with PI(4,5)P_2_ is responsible for coupling the GPCR to the heterotrimeric Gα_s_βγ [45]. The binding to PI(4,5)P_2_ stabilizes the GPCR and stimulates GTP hydrolysis [45]. Further work has shown that PI(4,5)P_2_ can stabilize the oligomeric state of adenosine A2A, which may lead to multi-output signaling complexes [46].

GPCR signaling can be attenuated in two different ways: (1) receptor molecules can be internalized, thereby slowing or stopping further ligand binding and the resulting cell signaling [47] or (2) the binding to G-proteins can be inhibited [47]. Both of these steps can be accomplished by arrestins [47] Arrestins bind to GPCRs and inhibit their interaction with G-proteins as well as mediate their internalization [47]. Recent work has shown that β-arrestin interacts with neurotensin receptor 1 [48]. Furthermore, this interaction is facilitated by a PI(4,5)P_2_ specific interaction with the C-lobe of β-arrestin and the TM cytosolic loops [48]. Other work has expanded on this and shown that the β-arrestin-GPCR complex requires PI(4,5)P_2_ to be fully stabilized [49]. Additionally, the β-arrestin interaction with the PM may actually increase PI(4,5)P_2_ levels to further facilitate GPCR internalization [50]. Thus, PI(4,5)P_2_ is involved in both the stabilization of the GPCR-G-protein signaling complex as well as the GPCR-β-arrestin signaling termination complex. In these ways PI(4,5)P_2_ is absolutely essential to the entire process of GPCR signaling, even when not specifically coupled to PLC or PI3K.

## PM transport

### Vesicular traffic

PI(4,5)P_2_ has a key role in recruiting proteins to the PM for the initiation and regulation of both exo- and endocytosis [3,7]. Exocytosis is a highly regulated process that leads to the release of intracellular components through the PM to the extracellular space, and/or the delivery of new membrane [3,7,51]. The three basic steps for exocytosis are vesicular docking, priming and fusion [51]. Docking involves the recruitment of vesicles and their tethering to the inner leaflet of the PM [51]. The exocyst complex of proteins is essential for this tethering process, specifically allowing tethering of the soluble N-ethylmaleimide-sensitive factor attachment protein receptors (SNAREs) [52,53]. The exocyst complex consists of two independent subcomplexes; subcomplex-1 is made up of Exoc1–4 and subcomplex-2 is made up of Exoc5–8. A majority of the exocyst complex is present on the vesicular surface, and when in proximity to the PM this complex interacts with Exoc1 (Sec3 in yeast) to form the full exocyst complex [52]. Exoc1 interacts with the PM initially due to an interaction with PI(4,5)P_2_ and a PM anchored SNARE protein [52–55]. After this initial interaction, another exocyst protein, Exoc7 (Exo70 in yeast), is now able to interact with PI(4,5)P_2_ at the PM as well, stabilizing the interaction of the vesicle with the PM and inducing membrane curvature [52,53,56].

The next step, priming, is responsible for the maturation of PM tethered vesicles [51]. This was in fact the first step to be recognized as PI(4,5)P_2_-dependent [57]. Munc13 and Ca^2+^-dependent activator protein for secretion (CAPS) are both required for the transition between docking and priming of vesicles [51]. Specifically, Munc13 and CAPS are involved in the formation and stabilization of the SNARE complex once they are localized with the PM [51,53]. Munc13 is recruited to the PM in a Ca^2+^-dependent manner, but once at the PM it interacts with and is stabilized by PI(4,5)P_2_ [51,58]. CAPS contains a PI(4,5)P_2_ binding PH domain which allows for its recruitment to the PM [58,59].

Finally, fusion allows for the vesicle to merge with the PM to release contents [51]. Components of the SNARE complex include syntaxin-1 and synaptosomal-association protein of 25 kDa (SNAP-25). Syntaxin-1 is a TM protein localized to the PM and containing a juxtamembrane polybasic domain that forms PI(4,5)P_2_-dependent clusters at sites of membrane fusion [60]. SNAP-25, on the other hand, localizes with the PM due to a palmitoylation motif [61], though a polybasic stretch is required to target the protein to the PM via PI(4,5)P_2_ for palmitoylation to occur [62]. Because of its myriad requirements, the loss of PI(4,5)P_2_ at the PM results in dysregulation of exocytosis, hindering this process at all steps [58,63,64].

Opposite of exocytosis, endocytosis is the process of moving cargo from the extracellular surface into the interior of the cell or retrieving PM components. PI(4,5)P_2_ has been shown to play a key role in clathrin-independent endocytosis [65,66] and phagocytosis [67]. Clathrin-mediated endocytosis (CME) is the best-characterized mechanism of endocytosis with a strict requirement for the lipid [68]. Fundamentally, CME relies on the recognition of cargo proteins on the cytosolic leaflet of the PM by adapter proteins, which then stimulate the assembly of the clathrin coat for vesicle budding. A requirement for PI(4,5)P_2_ in membrane cargo binding was recognized early on for the major PM adaptor complex, AP-2 [69]. Several additional clathrin adapter proteins have since been recognized as being recruited by PI(4,5)P_2_, including epsin [70] and AP180 [71]. In addition to being a simple membrane anchor, PI(4,5)P_2_ also seems to play a pivotal role in the allosteric activation of AP-2 complexes on the membrane [72,73].

In the final stage of CME, the budding clathrin-coated vesicle is separated from the PM via the constriction of the neck due to the action of dynamin [68]. Dynamin is recruited to the PM due its interaction with PI(4,5)P_2_ [74]. Because of these central roles, loss of PI(4,5)P_2_ from the PM completely blocks clathrin-mediated endocytosis [63,75–79]. Such a potent role for PI(4,5)P_2_ in clathrin-coated structure formation is emphasized by the fact that recruitment of PI(4,5)P_2_ 5-OH phosphatases like synaptojanin-1 and OCRL are essential for the uncoating process after vesicles bud [80–82].

### Selective permeability of the membrane

The presence of ion channels and transporters in the PM conveys the ability for selective permeability to ions and small molecules, and also confers the capacity for electrical excitability in neural and contractile tissues. The first indications for regulation by PI(4,5)P_2_ came with the demonstration that both the sodium/calcium exchanger (NCX) and ATP-sensitive potassium channels (*K*_ATP_) required the lipid to operate in cardiac myocytes [83]. Since then, additional membrane transporters have been shown to require PI(4,5)P_2_ for assembly and function, including the serotonin [84] and dopamine [85] transporters along with the PM calcium ATPase [86] and the epithelial sodium/proton exchanger, NHE [87].

The number of ion channel classes discovered to be regulated by PI(4,5)P_2_ has exploded. They include certain members of the voltage-gated (*K*_v_), inwardly rectifying (*K*_ir_) and calcium-activated potassium (*K*_Ca_) channel families, voltage-gated calcium channels, PM-localized transient receptor potential (TRP) channels, epithelial sodium channels (ENaC), cyclic nucleotide-gated channels (CNGs), purinergic-regulated P2X channels and calcium-activated chloride channels [5,88]. The crystal structure of a *K*_ir_ channel in complex with PI(4,5)P_2_ revealed a novel paradigm for the lipid's activation of membrane proteins: the acyl chains interact non-selectively with the TM helices of the channel, whereas the inositol headgroup binds selectively to the cytosolic domain, essentially pulling the domain into close contact with the pore helices and partially opening the ion conductance pore [89]. This highlights another fundamental mechanism of PI(4,5)P_2_ action: allosteric regulation of a protein (Figure 3C). For the most part, channels are activated by PI(4,5)P_2_ interactions, which have been proposed as a mechanism for restricting their activity to the PM, avoiding leakage during trafficking steps [90]. However, there are also examples of negative regulation of channels by PI(4,5)P_2_ binding [88], sometimes on the same channel that is also positively regulated by the lipid [91].

Regulation of ion channel activity is also a new paradigm for PLC regulation of PI(4,5)P_2_ function. Here, rather than generating second messengers, PLC removes PM PI(4,5)P_2_ to modulate channel activity. This was first described for M-current in neurons. M-current is mediated by *K*_ir­_, hence is inhibited by PLC-mediated PI(4,5)P_2_ depletion [92]. Since the M-current ordinarily buffers resting potential, the neurons themselves become more excitable. On the other hand, PI(4,5)P_2_ hydrolysis (and associated acidification) by PLC directly activates TRP channels in *Drosophila* photoreceptors [93]. We would underscore, however, that setting aside these specific physiological examples, most instances of PLC activation do not lead to substantial depletion of PI(4,5)P_2_ (see for example [10]). Most examples in the literature where PLC leads to depletion of PI(4,5)P_2_ rely on the over-expression of PLC-coupled receptors and/or the application of non-physiological, pharmacologically saturating concentrations of agonist. Even when PI(4,5)P_2_ depletion does occur, cellular systems possess machinery for its rapid resynthesis. Failure of PI(4,5)P_2_ resynthesis can be catastrophic. For example, in the *Drosophila* photoreceptor, failure to regenerate the lipid leads to detachment of the cytoskeleton (discussed in the next section) and degeneration of the photoreceptor cells [94].

Collectively, PI(4,5)P_2_ synthesis is necessary for maintaining ion and solute homeostasis in a variety of excitable and non-excitable tissues. Indeed, failure to activate NHE after PI(4,5)P_2_ breakdown by the *Salmonella* effector SopB is a primary reason why fluid uptake is blocked in the gastrointestinal epithelia during food-born infections, leading to diarrhea and dysentery [95].

## Structure and organization of the membrane

### Cytoskeleton

The actin cytoskeleton regulates cellular processes ranging from endocytosis to cell motility to cytokinesis, and it is tightly associated with the membrane [2,96]. Control of these dynamic cellular processes by actin relies on the fact that the actin cytoskeleton is itself remarkably dynamic [2,96]. PI(4,5)P_2_ is a central player in regulating these dynamics.

One of the first proposed roles for PI(4,5)P_2_ acting as more than a substrate for PLC was its ability to dissociate profilin-G-actin complexes, effectively nucleating actin polymerization on the PM [97]. Furthermore, F-actin severing by another abundant actin regulatory protein, gelsolin, was shown to be blocked by PI(4,5)P_2_ [98]. Finally, the actin-severing protein cofilin was shown to be inhibited by PI(4,5)P_2_ [99,100], and so can be released from the membrane after PI(4,5)P_2_ hydrolysis to sever actin filaments [101]. Therefore, PI(4,5)P_2_ was established as a pro-actin filament assembly signal. It should be noted, however, that the interaction of factors such as profilin and cofilin with PI(4,5)P_2_ is very low affinity, with a relatively small fraction of these proteins PM bound at physiological lipid levels [102]

Actin filaments can be capped, at barbed ends, by the capping protein CapZ [103–105] to prevent both actin assembly and disassembly [2,96]. CapZ can be removed from actin filaments due to an interaction with PI(4,5)P_2_, thus allowing for either PM actin assembly or disassembly [103–105]. Neuronal Wiskot–Aldrich syndrome protein (N-WASP) is recruited to the PM via an electrostatic interaction between the negatively charged inner leaflet, due in part to PI(4,5)P_2_, and a polybasic domain in N-WASP [106]. This interaction is much tighter than for cofilin or profilin. However, it is still dynamic enough for localization to be driven by dissociation from PI(4,5)P_2_, rather than by slow lateral diffusion of N-WASP with the lipid [102]. Once localized at the PM, N-WASP activates the actin-related protein 2/3 (Arp2/3) complex [107,108], which then nucleates daughter filaments from existing mother filaments to facilitate the branching of actin [96,109,110].

The actin cytoskeleton is closely linked to the PM by several proteins [2,96]. One such protein family is the ezrin, radixin, moesin (ERM) family of proteins, which like their red blood cell counterpart, band 4.1, all contain a FERM domain. In fact, the PI(4,5)P_2_-band 4.1 interaction was one of the first functions for intact PI(4,5)P_2_ to be defined [111]. FERM domains bind to PI(4,5)P_2_ with high affinity [102,112–115], triggering the unmasking of other binding sites in the ERM proteins which allow them to interact with F-actin or intracellular adhesion molecules (ICAMs) [112–115]. Another protein family linking both actin and microtubules to the PM are septins [2,116]. Septins have been shown to interact with PI(4,5)P_2_ via a polybasic domain that is separate from their actin/microtubule binding N-terminal domains [116,117]. Spectrins are the last family of proteins which similarly interact with actin and the PM [2,96]. Specifically, β-spectrin contains a PH domain which has been shown in bind PI(4,5)P_2_, allowing for PM association [118–120].

Cellular adhesion to the extracellular matrix (ECM) is another pivotal function of the cytoskeleton. Focal adhesions (FAs) are made up of a complex network of proteins that link the actin cytoskeleton to the ECM. The physical interaction with the ECM is facilitated by the extracellular portion of integrins [121]. The intracellular portion of integrins then generates the integrin signaling layer [121]. This layer contains integrins, paxillin, focal adhesion kinase (FAK) and talin [121]. Atop this is the force transduction layer, composed of talin, VASP and vinculin [121]. And finally, the actin regulatory layer is made up of VASP and α-actinin, attaching to bundled actin filaments. Each of these layers contains at least one PI(4,5)P_2_ interacting protein: FAK [121–123], talin [112,113,124–126], vinculin [124,127–130] and α-actinin [127]. PI(4,5)P_2_ is known to function as either an activator or a stabilizer for each of these proteins in each layer of the FA; thus the lipid seems to be a crucial factor in activating these proteins for FA assembly.

Clearly, PI(4,5)P_2_ is essential to the integration of a functional cytoskeleton with the PM. Indeed, aberrant PI(4,5)P_2_ levels can lead to many changes in the organization or regulation of the actin cytoskeleton [131–134]. For example, ectopic accumulation of PI(4,5)P_2_ on endocytic structures can lead to the polymerization of actin ‘comet tails’ on endosomes, disrupting vesicular traffic in Lowe Syndrome [81,135]. Conversely, loss of PM PI(4,5)P_2_ leads to the detachment of the actin cytoskeleton and a collapse in membrane tension [136,137].

### ER-PM contact sites

Anchoring of the ER to the PM is a requisite for a variety of essential cellular functions, including ER calcium store filling, traffic of lipids to the PM and stress sensing [138]. These functions are mediated by classes of proteins that tether the two membranes, possessing both PM and ER anchoring domains. For PM anchoring, PI(4,5)P_2_ is a common molecule that serves as the binding partner, mediating the interaction of extended synaptotagmins (E-Syts) [139], oxysterol-binding protein-related proteins (ORPs) 5 and 8 [140,141] and the GRAMD2 but not GRAMD1 proteins [142]. Because of these interactions, PI(4,5)P_2_ is essential for store-operated calcium entry [143] and the accumulation of PM phosphatidylserine [141].

### Cell polarity

PI(4,5)P_2_ has recently emerged as a key regulator of cell polarity. Particularly, the Par-6 complex is a key determinant of apical–basal polarity across a variety of tissues in animals [4]. Par-6 recruits an atypical protein kinase C (aPKC) to apical domains, which phosphorylates and thereby inhibits apical PM recruitment of basolateral proteins such as Lgl, Numb and Miranda. The specific site of phosphorylation in these proteins is an amphipathic, polybasic domain that targets the proteins to the PI(4,5)P_2_-rich PM [144,145]. Phosphorylation neutralizes some positive charge in these domains, reducing electrostatic interaction with the membrane. Intriguingly, aPKC itself contains a polybasic, amphipathic pseudosubstrate region that is not phosphorylated. This can similarly target PM PI(4,5)P_2_, but is not exposed for membrane interaction in the context of full-length aPKC until an allosteric activation mediated by Par-6 at apical domains [146]. Therefore, PI(4,5)P_2_ appears to be a critical determinant of both apical and basolateral membrane identity through context-dependent interactions with binding partners.

## PI(4,5)P_2_: signal or cofactor for PM function?

Given the many PM PI(4,5)P_2_-dependent PM functions we have met so far, we can imagine what would happen to a cell unable to synthesize PI(4,5)P_2_: It will be unable to transmit signals coupled to PLC or PI3K. Many GPCRs coupled to andenylyl cyclases will also not function efficiently, and signaling through a wide range of small GTPases will be blocked as they no longer localize at the PM. The cell will be unable to secrete proteins via the secretory pathway and be deficient in its capacity to undergo all forms of endocytosis. Its actin cytoskeleton will not be tightly integrated with the PM, causing a collapse of membrane tension. It will also be unable to efficiently build contacts with the ECM or neighboring cells. Many key contacts with the ER will not form, compromising the cell's capacity to maintain PM lipid and ER calcium homeostasis. And if it's a polarized cell, it will struggle to maintain polarity determining complexes. Put simply, the cell will be completely kaput.

The genetics seem to support this thought experiment. Loss of PIP5K α and γ genes in mice prevents embryonic development [147]. Loss of just the PIP5Kγ isoform leads to perinatal lethality in both mice and humans [63,148,149], stemming from a drop in PI(4,5)P_2_ levels in the nervous system [63]. Obviously, PI(4,5)P_2_ is an essential molecule to support animal life at the cellular level. On the other hand, several diseases are associated with failures to degrade PI(4,5)P_2_, mainly from loss-of-function mutations in INPP5 phosphatases (Figure 2) [150–155]. While these mutations do not preclude early development in the same way that loss of the kinases does, the phenotypes of these diseases are all devastating and lead to early mortality. So it seems in the case of PI(4,5)P_2_, there can be too much of a good thing. This also shows us that supporting the PM functions we have explored is more than just assuring that there is enough lipid for proteins to interact with; the levels must be carefully controlled, and accumulations can have adverse phenotypic consequences in their own right.

The shear diversity of PM function regulated by PI(4,5)P_2_ poses a conundrum in the context of the wide variety of phenotypes associated with aberrant PI(4,5)P_2_ metabolism: these can effect a range of tissues and cause quite disparate diseases, from problems with kidney filtration to neurodegeneration to muscular dystrophy [150,152–155]. Tissue-specific enrichment of the INPP5s may go a long way to explaining restriction of these diseases to specific organ systems, but the fact that patients survive to adulthood suggests that the phenotype is not driven by the total failure of all PI(4,5)P_2_-regulated PM function in the affected cell types. This poses a larger question: how is PI(4,5)P_2_ coupled to individual PM functions in health and disease?

The provenance of our understanding of PI(4,5)P_2_ function is firmly rooted in cell signaling, with its initial characterization as substrate for PLC and later PI3K signaling (Figure 1). The discovery that the lipid can also modulate actin polymerization led to the idea that PI(4,5)P_2_ is a second messenger in its own right. It follows that although cells maintain relatively stable PI(4,5)P_2_ levels, nanoscopic changes in its concentration may modulate individual functions that we describe above; the cacophony of these myriad signals then averages out to the global steady state [156]. Disruption of specific nano-scale pools of PI(4,5)P_2_ after disruption of individual phosphatases may then explain the restricted phenotypes associate with loss-of-function.

We think that such a view of PI(4,5)P_2_ as an extremely busy second messenger is unlikely to be correct. Firstly, empirical measurements have shown that many phosphoinositide effectors have dissociation rate constants in the millisecond to second-time scale [102,157,158], so effector-bound lipids are likely to be in rapid dynamic equilibrium with unbound lipids. Secondly, and most crucially, diffusion of lipids like PI(4,5)P_2_ on the inner leaflet of the membrane is rapid, ranging from 0.1 to 1 µm^2^/s [157,159–162]. This estimate takes the slowing effect of the cortical membrane cytoskeleton into account; inside cytoskeletal corrals, diffusion is an order of magnitude faster [163,164]. Therefore, even if we grant a ‘nanodomain’ a relatively generous diameter — say *r =* 100 nm, as found in clathrin-coated structures — at the lower limited of diffusion, it will take ∼80 ms for a PI(4,5)P_2_ molecule that is released from an effector to diffuse away (from *r* = √4*Dt*/*π*, where *D* is the diffusion coefficient [165]). Coupled to rapid dynamic exchange, it would be extremely difficult for cells to maintain localized PI(4,5)P_2_ accumulation on the time scales necessary for the minute-long processes it regulates. Local generation of lipid is likely to ‘spill out’ to neighboring complexes, producing unwanted regulation. Clustering of PI(4,5)P_2_ can be induced and maintained by electrostatic bridging between the negatively charged phosphates by polybasic peptide stretches in proteins [60,166] or by multivalent cations [167]. This could certainly generate different interaction properties of such clusters, which might favor the binding of polyvalent, non-specific effectors over stoichiometric binders [168]. However, reservation of such nanoscopic lipid clusters for the select physiological function would require either precise tuning of effector affinity to a narrow range of cluster density, or the presence of effector proteins in the cluster that define function through additional protein–protein interactions. In this latter case, note that it would not really be the PI(4,5)P_2_ that defines the function of the cluster, rather the specific ions or proteins inducing their formation — and thus playing the messenger role. In any case, in terms of PI(4,5)P_2_ in intact animal cells, several groups have found no evidence of nanoscopic PI(4,5)P_2_ accumulation [13,169], and others have found sample preparation artifacts that artificially induce them [170,171].

If PI(4,5)P_2_ is a lousy second messenger, what is it good at? We prefer to think of it in the terms that Hilgemann proposed for ion channels: ‘*In terms of cellular homeostasis, differences in the levels of PIP_2_ in internal compared with surface membranes may help to control the activity of proteins as they progress through the secretory pathway to the surface membrane*’ [90]. We would expand this to cover the full gamut of PI(4,5)P_2_-regulated function: PI(4,5)P_2_ serves as a necessary cofactor to activate cellular processes selectively at the PM. In other words, it is much more signpost than signal. Under this model, PI(4,5)P_2_ is a ‘master regulator’ of all PM functions, i.e. it is a common factor required for each. Spatial and temporal regulation of these processes (beyond simple activation by PI(4,5)P_2_ at the PM) is then driven by the many other, disparate signaling mechanisms that regulate them individually.

If this is the case, why do diseases and experimental manipulations that cause aberrant PI(4,5)P_2_ levels lead to such diverse phenotypes? Why do they only effect limited subsets of PM function? We think a clue comes from studies of cytoskeletal regulation: the effective PI(4,5)P_2_ affinity of different actin- binding proteins varies by two orders of magnitude [102]. Under such a paradigm, moderate changes in lipid levels (such as those caused by the loss of an individual INPP5) are unlikely to affect high-affinity processes (in this case, anchoring of cortical actin by ERM proteins), whereas those with low affinities may be drastically altered (e.g. *de novo* actin polymerization). The extent to which this is true will require a more quantitative analysis of PI(4,5)P_2_ affinity by its effectors. This is a daunting challenge, given that effectors generally interact with each other and other modulators, generating very high avidity complexes in the cellular milieu. Nonetheless, probing changes in different PI(4,5)P_2_-regulated processes under conditions of pathological PM PI(4,5)P_2_ alterations could be transformative; it would illuminate the phenotypic specificity of diseases driven by them — a crucial first step in formulating any new clinical intervention.

In conclusion, there is a vast array of PM function regulated by PI(4,5)P_2_. We have attempted here to convey the breadth of this function, though we have by no means been exhaustive. A cursory glance of the timeline in Figure 1 shows that the discovery of functions associated with the lipid has been accelerating over the last decade. We would predict that we do not yet have a complete list of PI(4,5)P_2_-dependent functions. The future is full of surprises.

**Perspectives**

- PI(4,5)P_2_ is an abundant and crucial regulator of PM function.
- Originally discovered as a source of second messengers for the PLC and PI3K signaling pathways. Now, the intact lipid is known to be required for the activation of PM transport, signaling and structural functions in its own right.
- Detailed mechanistic studies show how the lipid can recruit proteins to the membrane by stereo-specific or non-specific electrostatic interactions. Additionally, the lipid can induce allosteric activation of binding proteins.
- A key question moving forward is how one class of molecule regulates so many parallel processes. Specifically, the extent to which this is controlled by localized PI(4,5)P_2_ signaling versus activation thresholds of individual processes for a plasma membrane wide PI(4,5)P_2_ concentration needs to be deduced. This will be crucial to understanding the causes of diseases resulting from aberrant PI(4,5)P_2_ metabolism.

## Abbreviations

aPKC: atypical protein kinase C
CAPS: Ca^2+^-dependent activator protein for secretion
CME: clathrin-mediated endocytosis
CNGs: cyclic nucleotide- gated channels
ECM: extracellular matrix
ENaC: epithelial sodium channels
ERM: ezrin, radixin, moesin
E-Syts: extended synaptotagmins
FAK: focal adhesion kinase
Fas: focal adhesions
GPCRs: G-protein-coupled receptors
ICAMs: intracellular adhesion molecules
NCX: sodium/calcium exchanger
N-WASP: neuronal Wiskot–-Aldrich sSyndrome protein
ORPs: oxysterol-binding protein-related proteins
PI3Ks: phosphoinositide 3-kinases
PLC: phospholipase C
PM: plasma membrane
SNAREs: soluble N-ethylmaleimide-sensitive factor attachment protein receptors
TM: transmembrane
TRP: transient receptor potential
Arp2/3: actin- related protein 2/3
